# Treatment failure in suspected community-acquired pneumonia revealing a pulmonary abscess

**DOI:** 10.1177/2050313X261456973

**Published:** 2026-06-15

**Authors:** Sindhuja Earagolla, Noah Karnath, Sravya Borra, Bernard Karnath

**Affiliations:** 1 John Sealy School of Medicine, University of Texas Medical Branch, Galveston, TX, USA; 2 Department of Medicine, Albert Einstein College of Medicine, Bronx, NY, USA; 3 Department of Internal Medicine, University of Texas Medical Branch, Galveston, TX, USA

**Keywords:** community-acquired pneumonia, chest X-ray, CT thorax, diagnostic imaging, pulmonary abscess

## Abstract

Community-acquired pneumonia (CAP) is a leading cause of hospitalization and mortality, with *Streptococcus pneumoniae* as a leading cause. Chest radiography, the standard initial imaging modality, can miss early complications, which lead to delays in diagnosis and treatment. Computed tomography (CT) provides greater detail in identifying complications. We report a 67-year-old male with chest pain who was treated in an outpatient setting with oral antibiotics for suspected CAP; however, he experienced clinical deterioration, including fever, cough, weight loss, and worsening respiratory symptoms. Repeat chest X-ray revealed increased lung opacities, prompting a CT thorax, which revealed a large pulmonary abscess not visualized on prior imaging. Failure of outpatient management required inpatient admission with intravenous antibiotics and CT-guided percutaneous drainage. A total of 450 mL of purulent fluid was drained, and cultures grew *Streptococcus intermedius*. The patient improved and was given targeted antibiotics. This case highlights the importance of considering less common pathogens such as *S. intermedius* in patients with prolonged symptoms or failure of outpatient therapy. CT imaging should be considered in patients who fail to improve, thus expediting diagnosis and guiding management. *S. pneumoniae* typically causes acute pneumonia with lobar consolidation. *S. intermedius* often causes necrotizing abscesses and empyema, often with a longer, more subacute presentation. Differentiating the two on initial presentation is quite difficult, as in our patient’s case.

## Introduction

Community-acquired pneumonia (CAP) is a frequent cause of hospitalization, especially in patients who are older or have comorbid illnesses. In cases of severe CAP, pulmonary complications can occur, such as lung abscess, empyema, pleural effusion, acute respiratory distress syndrome, and necrotizing pneumonia.^1^ The diagnosis of CAP can be missed even with early chest radiography (CXR).

Currently, the gold standard of radiographic imaging is CXR. However, chest radiographs can miss lung nodules, lung cancer, pneumonia, pneumothorax, pleural effusions, and more abnormalities.^2^ Several studies have shown that computed tomography (CT) scans are useful when chest radiographs show unclear results. One study showed how in half the patients diagnosed with CAP, the chest radiograph was negative or unclear, but a CT led to the diagnosis.^3^ One case report shows how a chest radiograph missed the detection of a pneumonia complication, an abscess, while a subsequent CT detected the presence.^4^ Another study described a case of a large lung abscess that was initially misdiagnosed and treated as an empyema on chest radiograph until correctly diagnosed by CT.^5^ The literature shows how CT-only pneumonia – pneumonia that is negative on chest radiograph – is a common finding, and symptoms, disease progression, and response to therapy are similar to those of chest radiograph-positive pneumonia.^6^ A CT scan led to a more accurate diagnosis and changed decision-making in terms of initiation of antibiotics versus discontinuation in patients or discharge.^7^

Our case highlights the clinical value of timely CT imaging in identifying complications of CAP that may not be apparent on initial CXR. Our case also underscores the importance of considering less common pathogens such as *S. intermedius* in patients with prolonged symptoms or failure of outpatient therapy. Whereas *S. pneumoniae* typically causes acute pneumonia with lobar consolidation, *S. intermedius* causes necrotizing abscesses and empyema. Differentiating the two on initial presentation can be difficult.

## Case presentation

We report a case of a 67-year-old male with a significant past medical history of hypertension, hyperlipidemia, pericarditis, and nonobstructive coronary artery disease. He presented to the emergency department with a chief complaint of acute, sharp, and left-sided chest pain. The pain was exacerbated when lying down, and tender points were identified on the left chest wall. The patient was playing golf the day before onset and described feeling very sore and winded the next day, which progressed throughout the week. He denied fevers and chills at initial presentation.

**Figure 1.**
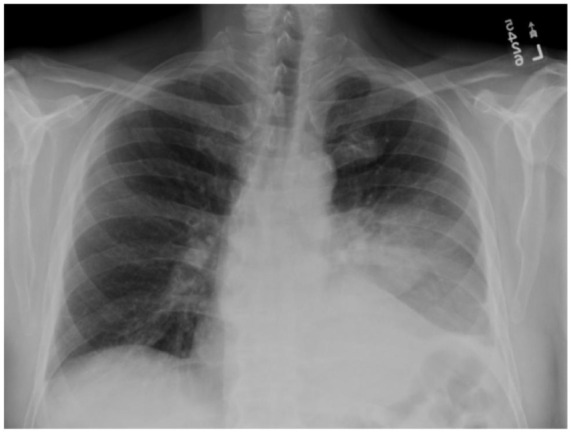
Initial CXR in the hospital showing left lung airspace opacities suspicious for CAP. CXR: Chest radiography; CAP: Community-acquired pneumonia.

On initial evaluation, the patient presents with a blood pressure of 129/78 mmHg, pulse of 60 beats/min, respiratory rate of 18 breaths/min, temperature of 37.1°C (98.8°F), and an oxygen saturation of 99% on room air. The initial laboratory evaluation included normal cardiac enzymes (troponin I = 0.006 ng/mL), EKG without ST elevations, and chest X-ray. CXR revealed a small left-sided pleural effusion (Figure 1), and chest pain was assessed to be pleuritic in nature. Cardiology was consulted due to suspected pericarditis because of the patient’s past medical history; however, transthoracic echocardiogram revealed a normal left ventricular ejection fraction with no wall motion or valvular abnormalities and no pericardial effusion. The patient was treated for musculoskeletal pain/costochondritis and was discharged on ibuprofen 400 mg three times daily, and pantoprazole 40 mg for 1 week.

The patient reported that the pain had gotten worse at his follow-up primary care physician visit the next day. He also developed a productive cough with thick yellow sputum and had a fever and chills the previous night. Laboratory evaluation at this time showed a procalcitonin level of 0.36 ng/mL. Treatment for suspected CAP was initiated and managed as an outpatient with oral doxycycline 100 mg and amoxicillin-clavulanate 875–125 mg to provide coverage for typical and atypical CAP pathogens.

**Figure 2.**
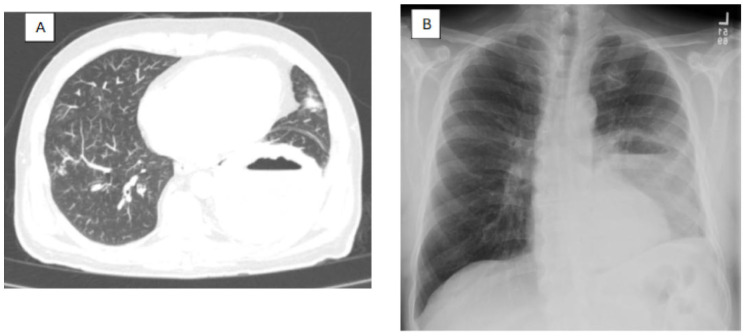
(a) Repeat CXR shows large air-fluid levels in the left lower hemithorax. The left hemidiaphragm is elevated, likely secondary to the mass effect of a cavitary lesion. Trace left pleural effusion or pleural thickening is noted. No other evidence of pneumothorax or overt congestive heart failure. (b) CT Thorax with contrast showed a large air and fluid collection in the left lower lobe with a thickened enhancing wall measuring about 9.5 × 10.4 × 12.7 cm. CXR: Chest radiography; CT: Computed tomography.

Despite therapy, the patient returned in 1 week with persistent chest pain, non-productive cough, night sweats, and chills. On physical examination, there were diminished breath sounds and dullness to percussion in the left lower lung. Repeat CXR (Figure 2(a)) showed large air-fluid levels in the left lower hemothorax, raising concern for a complicated pulmonary or pleural process. Given the failure of outpatient management, the patient was admitted for inpatient care immediately, and a CT thorax with contrast (Figure 2(b)) was ordered to further characterize the lesion and guide management. Imaging revealed a large air and fluid-containing collection in the lower left lobe with a thickened enhancing wall, suggesting a large pulmonary abscess.

**Figure 3.**
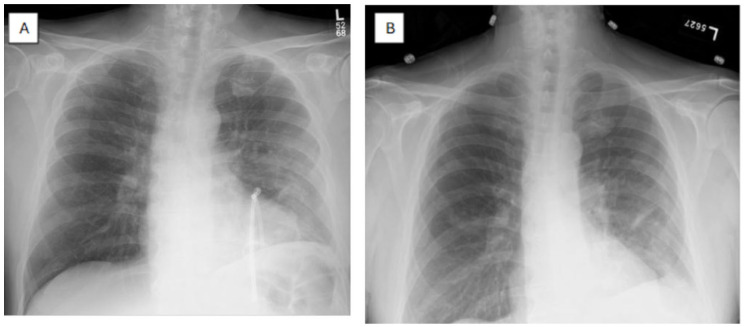
(a) CXR showing left basilar pleural drain appears to be kinked. Small residual left pleural effusion. There is upward displacement of the left hemidiaphragm, likely due to the adjacent pulmonary abcess. (b) Follow up CXR shows minimal pleural fluid remaining after removal of the left chest tube with subsegmental atelectasis in the left lung base. The right and upper left lungs are clear, and the heart and mediastinum are unremarkable. CXR: Chest radiography.

The patient was admitted to the hospital for drainage of the abscess, and 450 mL of purulent fluid was drained after a chest tube was placed (Figure 3(a)). Fluid cultures revealed 3+ *Streptococcus intermedius*, and laboratory evaluation showed a procalcitonin level of 0.22 ng/mL. The patient was discharged on oral amoxicillin-clavulanate 875 mg twice daily for 30 days. Follow-up CXR (Figure 3(b)) showed an interval decrease in abscess with residual consolidation. During hospitalization, the patient also developed anemia of chronic disease.

## Discussion

CAP, as previously established and as observed in the presented case of this patient, is a frequent cause of severe illness that can potentially lead to hospitalization and can present with a complex course and various complications.^3^ As such, it is important to evaluate the efficacy of tests, imaging, and medication that are used in the workup and treatment for cases of CAP to increase prevention of such complications.

In this case, initial outpatient management was considered given the patient’s hemodynamic stability and absence of radiographic evidence of complicated infection. Although pleuritic chest pain was present, the overall clinical picture did not initially suggest complicated pneumonia requiring hospitalization, and alternative etiologies due to musculoskeletal or cardiac origins were considered. Empiric dual antibiotic therapy with amoxicillin-clavulanate and doxycycline was selected for broad coverage against typical (e.g. *S. pneumoniae* and *H. influenzae*) and atypical (e.g. *Mycoplasma* and *Chlamydia*) bacteria. Despite this, the patient showed signs of clinical worsening, indicating progression of disease and the need for inpatient evaluation.

Traditionally, the chest radiograph has been regarded as the first-line diagnostic imaging study to be done for evaluating a potential case of CAP.^5^ However, CT imaging has recently seen a rise in usage as an alternative, or concurrently, to CXR.^7^ As seen in this case, selective use of both imaging modalities based on clinical presentation and response to therapy may be more beneficial to the patient than relying on a single technique. Importantly, this case also highlights the diagnostic uncertainty that can occur during initial clinical evaluations in the emergency department evaluations of CAP, where image findings can be subtle or nonspecific and require clinical reassessment.

In this patient’s case, initial chest radiographs failed to indicate the severity of the complications of his pneumonia. However, the CT scan that was performed following the visualization of air-fluid levels on radiography fully indicated the scope of the lung abscess that the patient had developed. CT imaging was not performed initially due to low suspicion for complicated pneumonia and was reserved for later when radiographic progression and treatment failure raised concern for abscess formation.

The chest radiograph remains regarded as the first-line imaging study for CAP. The ease of use and availability of the test, in addition to the relatively low harm posed because of radiation exposure, make it a simpler choice for imaging modality.^6^ In this patient’s case, during his initial, uncomplicated presentation, the low cost and ease of use were the likely reasons to perform this imaging study. CXR has a strong use as an initial screening tool, but it may be worth considering a concurrent imaging modality in more complicated cases, where the etiology of the condition is less certain and complications are more likely. Radiography also has some issues regarding the interpretation of images, which can result in mistakes and missed findings. In addition to abscesses, as seen in this case, lung nodules, pneumothorax, hilar masses, lymphadenopathy, and metastasis are other major findings that are prone to being missed or mistaken.^4^ This is another reason to consider using additional imaging studies to ensure that no complications are missed. In addition, cognitive factors such as anchoring bias may contribute to premature diagnostic closure in the emergency department setting, particularly in patients with overlapping cardiopulmonary symptoms and prior relevant cardiac history, such as this patient’s history of presumed viral pericarditis.

In a case report describing a similar presentation to the one described here, CXR was inconclusive when the patient returned with worsening symptoms, and a CT was needed to be performed to conclude that the patient had an empyema rather than an abscess.^8^ Another case series described similar outcomes requiring CT scans to be performed following unclear radiographs, with the authors noting that the sensitivity and specificity of CXR for distinguishing empyema from abscess are lacking in comparison to the CT scan.^9^ Furthermore, in another study, the CT scan revealed an infiltrate in 33% of patients who did not have one on chest radiograph and excluded pneumonia in 30% of patients with parenchymal infiltrate on chest radiograph.^7^ It is clear that the CT scan tends to show higher efficacy in diagnosing abscess. However, this does not mean that CT scans should be performed for every suspected case of CAP. Doing so could expose patients to unnecessary radiation and result in distraction from incidental findings. The exact threshold of when to perform a CT scan in the case of CAP is a matter of some debate, with some suggesting that it be used in a post-hospital setting for patients with high risk factors, such as increased age or smoking status.^7^ One study found that using a prediction score for patients suspected of having CAP resulted in being able to forgo performing a CT in at least half of all patients who presented.^10^

This patient’s case is also significant for the use of procalcitonin lab values in evaluating CAP. There is an established link between increased levels of procalcitonin and the diagnosis of bacterial pneumonia. For this reason, procalcitonin is commonly used as a diagnostic biomarker for evaluating CAP.^11^ In this patient’s case, at his initial visit with his primary care physician, he had elevated laboratory values for procalcitonin, causing suspicion for CAP, leading to the prescription of amoxicillin-clavulanate that day. When the patient was admitted for inpatient care following worsening of his condition, his levels of procalcitonin were once again elevated, and appropriately, following the treatment of his abscess, he was once again discharged with the appropriate antibiotic. In both instances during the patient’s case, the laboratory value for procalcitonin provided support for the diagnosis of bacterial CAP.

The causative organism being cultured as *Streptococcus intermedius* is another significant factor in this case. Streptococcus intermedius is a member of the *Streptococcus anginosus* group of bacteria, which consists of *S. anginosus*, *S. intermedius*, and *S. constellatus*. These organisms are often linked to cases of empyema and, occasionally, CAP.^12^ It can also cause cavitation and pleural effusion, as seen in this patient’s case. According to 1 study, *Streptococcus intermedius* was found in 54% of a group of 57 patients with a community-acquired pleural space infection, alongside *Fusobacterium nucleatum*.^13^ It is also important to note that *S. intermedius* is often a co-pathogen alongside anaerobes, which contributed to the choice of antibiotic therapy of amoxicillin-clavulanate in this case.^14^ A lack of improvement despite oral antibiotics, as in this case, can indicate a potential abscess. At that time, imaging would be indicated, which was done in this case. The repeat radiograph taken showed an air-fluid level, which led to the described workup regarding the patient’s abscess. In cases similar to this patient’s, one retrospective cohort study indicated that S. intermedius was the most common causative organism of empyema and CAP.^14^

Regarding the patient’s treatment, the patient’s abscess responded well to the second course of antibiotics, and he only required a chest tube for drainage of fluid. Therapeutic options for empyema include antibiotic therapy alone in early complicated cases, image-guided drainage, or thoracentesis for removal of fluid. Surgical intervention was not indicated in this case and is typically only indicated in the case of lung abscesses, which do not improve after 7–10 days of antibiotic treatment. According to the standard of care in the Chest Journal, empyema is typically treated with direct chest tube drainage, whereas abscesses are treated with antibiotic therapy.^15^

Surgical intervention is typically reserved for patients who fail to improve clinically or radiographically with antibiotic therapy. Surgical intervention is used in about 10%–15% of cases refractory to non-operative management.^16^ Although it did not occur in the case of this patient, there is always concern for sepsis in the event of a widespread bacterial infection such as this. In the event of it occurring, the patient would be admitted to the MICU, but since the patient presented with normal vital signs just prior to hospitalization, he did not meet the criteria for sepsis. This can be determined with the qSOFA or NEWS score, for which the patient had a score of zero on both.^17^

To further address variability in imaging utilization, a structured decision framework for CT imaging can be considered. CT imaging may be appropriate when (1) clinical severity is disproportionate to radiographic findings, (2) there is failure or clinical deterioration despite appropriate antibiotic therapy, or (3) there is diagnostic uncertainty with initial imaging. This framework can support judicious use of radiographic imaging while balancing diagnostic accuracy with radiation exposure and resource utilization.

## Conclusion

Overall, this case illustrates the importance of performing a thoracic CT early or the selective use of thoracic CT in patients with atypical presentations of CAP or failure to respond to standard therapy. Current clinical practice recommends CXR as the first-line imaging study for all patients with subsequent CAP, followed by empiric antibiotic therapy. Clinical response should be reevaluated 48–72 h after initiation of therapy. If there is a lack of improvement, clinical deterioration, or suspicion for complications such as abscesses, escalation to CT imaging should be considered, as well as reconsideration of the original diagnosis.

Less common pathogens such as *S. intermedius* should be considered in patients with prolonged symptoms or failure of outpatient therapy. Follow-up imaging is helpful in detecting complications of pneumonia, such as a lung abscess in our patient’s case. CT imaging should be considered in patients who fail to improve. While *S. pneumoniae* typically causes acute pneumonia with lobar consolidation. *S. intermedius* often causes necrotizing abscesses and empyema, often with a longer, more subacute presentation. Differentiating the two on initial presentation is quite difficult, as in our patient’s case.
